# A peer-review decision support tool for research funding decision-making using the analytic hierarchy process

**DOI:** 10.1371/journal.pone.0350938

**Published:** 2026-08-12

**Authors:** Hasina Al Harthi, Maryam Al Nabhani, Sulaiman Al Sabei, Amira A-Amri, Shaima Al Hinaai, Sa’ada AlDhuhli

**Affiliations:** 1 Department of Training and Studies, Royal Hospital, Ministry of Health, Muscat, Oman; 2 Department of Fundamentals and Administration, College of Nursing, Sultan Qaboos University, Muscat, Oman; 3 Department of Operations Management and Business Statistics, College of Economics and Political Science, Sultan Qaboos University, Muscat, Oman; National Kaohsiung University of Science and Technology, TAIWAN

## Abstract

**Background:**

Research funding is a crucial driver of healthcare innovation and improved patient outcomes. However, the peer-review process utilised during funding allocation is often subjective, inconsistent, and prone to reviewer biases, leading to inefficiencies in selecting high-impact projects. This study aimed to develop an objective, transparent, and structured scoring system to improve research funding decisions.

**Methods:**

A mixed-methods approach was used, incorporating a scoping review, surveys, Delphi methodology, and the analytic hierarchy process (AHP). The scoping review identified funding criteria used by national and international agencies. A survey of researchers and decision-makers at the Royal Hospital in Muscat, Oman, gathered insights into funding priorities. Experts refined and validated criteria using the Delphi method, while weighted importance to each criterion as per the AHP was assigned through pairwise comparisons.

**Results:**

The study identified 10 key criteria for funding decisions: scientific merit, ethical standards, novelty and innovation, significance and strategic alignment, feasibility, impact and knowledge transfer, budget and cost-efficiency, team expertise and collaboration, sustainability, and partnerships in funding. Based on AHP weighting, the final model prioritised Ethical Standards (21.7%), Scientific Merit (19.1%), and Novelty and Innovation (16.7%) as the highest-weighted criteria, together accounting for more than half of the overall decision weight.

**Conclusion:**

The proposed scoring model provides a structured, evidence-based framework to enhance research funding decisions by enhancing consistency and transparency during peer review. It ensures alignment with institutional and national research priorities, while the inclusion of impact, feasibility, and sustainability criteria reflects a shift towards funding research that yields tangible societal and healthcare benefits. Future studies should evaluate implementation of the model in real-world settings and explore the role of artificial intelligence-driven decision-support tools to further refine research funding processes.

## Introduction

Efforts to strengthen evidence-based healthcare practice and decision-making have driven increased investment in research funding. However, this investment is only effective if it translates into meaningful impact and value [1]. To maximise research benefits, every stage of knowledge development plays a crucial role in ensuring efficiency, minimising waste, and enhancing impact, including funding decisions [1,2]. The effectiveness of health research is further shaped by the complexity of medical issues, the extended timeline required for basic science to yield clinical applications, and socioeconomic and political factors influencing funding priorities [3]. Additionally, the allocation of limited funds complicates the process. While peer review remains the standard for funding decisions, it often lacks precision and is subject to numerous challenges [4].

Despite these challenges, peer review in funding decision-making can be improved through innovative approaches. Streamlining review processes [5], leveraging technology [6], incorporating public participation [7], and establishing effective feedback mechanisms [8] can allow funding organisations to enhance the quality and efficiency of their decision-making processes. These improvements are essential to ensuring that research funding is allocated effectively, ultimately advancing scientific knowledge and improving health outcomes.

A major issue of the current peer-review system is ambiguity and inconsistency in applying evaluation criteria. Variations among funding agencies and individual reviewers lead to misalignment between applicant expectations and reviewer priorities [9,10]. The process often fails to capture the multifaceted nature of research quality, focusing narrowly on intrinsic research excellence while overlooking ethical considerations and societal relevance, which may disadvantage interdisciplinary and innovative proposals [10,11]. Reviewer biases, influenced by background and institutional affiliations, further distort evaluations, often favouring established researchers over emerging scientists [9,12]. Additionally, the lack of transparency in applying criteria and providing feedback undermines accountability and trust in the process [8]. The predictive validity of peer review is also debatable, as reviewer scores do not consistently correlate with the future impact of funded research [13,14]. Addressing these issues is crucial to improving the fairness and effectiveness of research funding decisions.

To reduce subjectivity in research proposal evaluation and enhance the selection of high-impact projects, a more robust scoring system is needed. This study aimed to develop a unified scoring framework to improve the peer-review process for research funding allocation at the Royal Hospital, a tertiary care facility in Muscat, Oman. By increasing transparency and ensuring consistent scoring among peer reviewers, the proposed system benefits both funders and researchers, ultimately supporting more equitable and effective research funding.

This study applies a structured multi-criteria decision-making approach based on the Analytic Hierarchy Process (AHP), informed by expert input, to develop a transparent and adaptable research funding decision-support tool. The methodological innovation lies in translating qualitative expert judgments into a quantitative, policy-relevant scoring framework tailored to institutional and national research priorities. The resulting model identified ethical standards, scientific merit, and innovation as dominant funding criteria and demonstrated stable prioritization under alternative weighting scenarios.

## Methods

### Research design and setting

This mixed-methods study was conducted at the Royal Hospital, a tertiary care facility serving the Omani population and providing specialised healthcare. The hospital also plays a key role in national medical education, training, and research. Between 2019 and 2024, healthcare providers at the hospital contributed to more than 743 published works [15].

### Data collection

**Fig 1:**
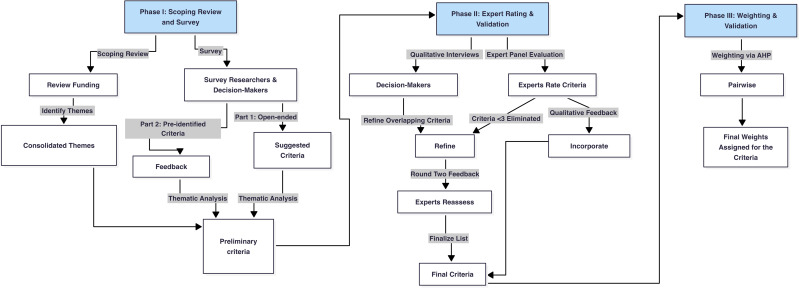
Three phases of the study.

The study comprised three main phases (Fig 1), using a mixed-methods approach that included a scoping review, surveys, Delphi methodology, and the analytic hierarchy process (AHP). The scoping review identified initial funding criteria, which informed a stakeholder survey to capture local priorities. These were refined through Delphi consensus, with AHP used to assign weights. Each phase built on the previous one, with outputs iteratively informing the next to refine and validate the criteria.

#### Phase I: Scoping review and survey.

A scoping review was conducted to identify criteria used by major national and international agencies. The criteria were consolidated into overarching themes. Subsequently, all staff with research experience or who were involved in funding decisions were invited to participate in an online survey to identify and evaluate funding criteria. No exclusion criteria were applied. Survey links were disseminated via research focal points in all hospital departments, official hospital messaging groups, and the in-hospital communication system. The survey remained open for two weeks from 20^th^ October 2024.

The survey comprised three sections. The first included an open-ended question inviting participants to propose key funding criteria. In the second section, participants were asked to review a structured list of criteria to prompt reflection and stimulate further input. The third section was an open-ended question asking participants to reflect on challenges in the peer-review process to inform criteria development and address practical barriers to improve funding decisions. Responses to the open-ended questions were analysed thematically through a systematic process: coding meaningful units, grouping codes into themes, and refining themes for consistency and relevance.

#### Phase II: Expert rating and initial validation.

On 4^th^ November 2024, a panel of 15 experts in healthcare, academia, and policy fields was convened to evaluate the preliminary criteria. Experts were purposively selected based on their dual roles as researchers involved in reviewing and scoring funding applications and/or as senior decision-makers responsible for research management, budget allocation, and sector strategy. Each criterion was rated for suitability on a 5-point Likert scale from 1 (strongly disagree) to 5 (strongly agree). Criteria with a median rating below 3 were eliminated. Experts also provided qualitative feedback to refine existing or propose additional criteria.

Subsequently, on 14^th^ November 2024, face-to-face interviews were conducted with three senior decision-makers with extensive research experience to develop the first draft of the final list of criteria. These interviews provided deeper insights into each criterion, highlighting their relevance in funding decisions, practical considerations that could impact the model’s weighting, and opportunities to merge overlapping criteria, improving the coherence of the final model. The revised criteria were then re-evaluated on 17^th^ November 2024 in a second round by the expert panel, who rated the relevance of each criterion on a 5-point Likert scale from 1 (not relevant) to 5 (highly relevant) and provided further feedback. The final list of criteria was developed based on this iterative process.

#### Phase III: Weighting and validation.

The AHP, a structured decision-making approach that quantifies subjective judgments, was applied to prioritize 10 predefined criteria for research funding decisions. Pairwise comparisons were used to generate weightings for each criterion based on relative importance [16,17]. Two formats were adopted. First, during an in-person workshop at the Royal Hospital in late November 2024, four members of the hospital’s Research Committee completed comparisons using the Saaty scale (1–9) [18], which captures the relative importance of one criterion over another. Second, a panel of three additional experts in clinical research, health strategy, and funding evaluation—who had participated in earlier study phases—were provided the AHP matrix during site visits and later submitted their responses via email within three days.

In both groups, each participant completed a reciprocal comparison matrix. Normalised weights were calculated by averaging values across rows, followed by a group average to represent consensus priorities. Consistency ratio was used to assess judgment reliability, with values below 0.10 indicating acceptable consistency. While several raters met this threshold, others showed moderate to high inconsistency; however, all inputs were retained to preserve diversity of perspectives, with the group averaging process mitigating individual variation. This participatory, multi-source approach enhanced the transparency, fairness, and policy relevance of the final weighted criteria.

Sensitivity analysis was conducted to assess the robustness of the prioritisation framework to plausible variations in stakeholder weighting preferences**.** One-way sensitivity analyses were performed by independently varying the relative weight of each criterion within a policy-relevant range while maintaining the overall hierarchical structure and total weight at 100%, and examining resulting changes in ranking. In addition, a multi-criterion scenario analysis was conducted to simulate an alternative policy emphasis through proportional redistribution of weights. Ranking stability across scenarios was interpreted as an indicator of model robustness.

### Ethical considerations

Ethical approval for this study was obtained from the Centre of Studies and Research, Ministry of Health, Oman, prior to data collection (#MOH/CSR/24/27981).

## Results

### Phase I: Scoping review

Thematic analysis was conducted of research funding priorities at three national institutions—the Ministry of Health, Ministry of Higher Education, Research and Innovation, and Sultan Qaboos University—and seven international institutions—the National Institutes of Health, UK Research and Innovation, the Australian Government Department of Health, the Southern African Research and Innovation Management Association, the Canadian Institutes of Health Research, the European Commission, and the Health Research Council of New Zealand.

Table 1 synthesises findings from the literature and institutional funding guidelines detailed in Appendix I and illustrates the consistency of core evaluation domains across funding contexts, which informed the theme construction in the proposed framework.

**Table 1:** Summary of funding evaluation criteria reported in the literature and institutional guidelines.

Funding body/context	Core evaluation themes	Key criteria emphasized
TRC (Oman)	Scientific quality, relevance, and feasibility	Novelty, literature review quality, methodology, team qualifications, relevance to national priorities, value for money
Sultan Qaboos University (SQU)	Intellectual merit, impact, capacity building	Scientific originality, expected impact, multidisciplinary approach, research team profile, capacity building
Ministry of Health (Oman)	Scientific merit, feasibility, impact	Clarity of proposal, novelty, methodology, feasibility, team capability, health system impact
NIH (USA)	Scientific and technical merit	Significance, investigator expertise, innovation, approach, environment, data management, ethics
UK Research and Innovation	Vision, approach, team capability	Research excellence, innovation, feasibility, risk management, impact, ethics, cost justification
Australian Government (Health)	Value and outcomes	Public benefit, value for money, feasibility, targeting priority populations
Southern African RIMA	Scientific impact and feasibility	Novelty, alignment with priorities, feasibility, literature quality, track record, collaboration
Canadian Institutes of Health Research	Merit, feasibility, impact	Research quality, interdisciplinarity, feasibility, impact, training and capacity development
European Commission	Excellence and impact	Ground-breaking research, ambition, feasibility, PI expertise, resource justification
Health Research Council of New Zealand	Rationale, design, impact, team	Scientific merit, innovation, impact, feasibility, capacity building, ethics

By reviewing and comparing criteria, common focus areas were identified and subsequently grouped into 17 categories.

Key categories included *Novelty and Originality*, assessing the introduction of new ideas and the proposal’s differentiation from prior work, ensuring that projects are genuinely innovative; *Literature Review and Background*, evaluating whether the proposal effectively justifies the research gap, grounding the research question in a comprehensive review of existing studies; and *Clarity and Coherence*, ensuring well-defined objectives and clear communication of the research approach, a crucial aspect for understanding the proposal’s goals and methodologies.

*Significance and Impact* covered the broader contributions of the research, considering the project’s potential contribution to scientific, societal, or economic spheres. *Methodology and Feasibility* assessed the study design’s robustness, ensuring that the approach, timeline, and budget were realistic and achievable. *Qualifications and Expertise of the Research Team* evaluated the investigators’ relevant experience and capacity to execute the project successfully, while *Research Environment and Resources* encompassed institutional support, infrastructure, and available facilities. *Interdisciplinary and Collaborative Approach* recognised the value of cross-disciplinary partnerships, particularly in tackling complex research questions, while *Alignment with National or Institutional Priorities* determined whether the proposal addressed key strategic objectives, a critical factor in funding decisions.

Furthermore, *Public Awareness and Knowledge Transfer* and *Research Capacity-Building* emphasised the project’s outreach, skill development, and infrastructure enhancement. Various other categories, such as *Budget Justification and Cost-Efficiency*, *Data Management and Ethical Standards*, and *Sustainability and Long-term Benefits*, assessed the practical and ethical dimensions of the project, ensuring responsible resource use and potential for lasting impact. Other categories identified during the scoping review included *Outcome Maximisation* and *Project Timeline and Management*, both of which examine the project’s potential to produce impactful results and manage operations efficiently.

### Phase I: Survey

A total of 96 respondents completed the survey, of which 37.5% were researchers, 24.0% were decision-makers, and 12.5% held both roles. Most of the criteria identified by survey respondents aligned closely with the thematic categories established during the initial scoping review. Frequently mentioned areas such as *Innovation and Novelty*, *Feasibility*, *Team Expertise*, and *Alignment with Institutional Priorities* reaffirmed the relevance and comprehensiveness of the original framework. This convergence suggests that the foundational themes derived from national and international sources were reflective of stakeholder perspectives.

Nonetheless, the survey also revealed additional considerations not previously captured. These included *Impact and Relevance*, with a focus on the alignment of research with emerging healthcare challenges and the specific needs of the local context. Respondents also highlighted *Resource Allocation*, emphasising the importance of balancing budget size with justification and anticipated outcomes. *Operational Readiness* was another emergent theme, reflecting the need for clearly defined implementation plans, realistic timelines, and risk mitigation strategies. These newly identified elements were incorporated to refine and strengthen the thematic framework. Their inclusion enhanced the applicability of the criteria by integrating frontline insights and ensuring the final set of themes reflected both established priorities and practical, context-specific considerations relevant to research funding decisions.

Respondents acknowledged challenges in balancing innovation with *Feasibility and Resource Allocation*. Resource limitations were cited as a significant constraint, forcing decision-makers to make difficult trade-offs between equally promising projects. Concerns were also raised about *Bias Mitigation in Decision-Making*, particularly regarding potential favouritism towards familiar researchers or conventional topics.

### Phase II: Expert rating

In round 1 of the panel, 13 out of 15 experts participated. Their feedback included insightful suggestions on incorporating additional criteria for funding decision-making, highlighting the importance of essential factors like practical impact, stakeholder engagement, and research outcome relevance (Table 2).

**Table 2:** Key factors identified by experts when prioritising funding for research projects in Round 1 (n = 13).

Factor	Average Likert score*	Considerations
Novelty and Originality	4.46	◦ Is the proposal introducing genuinely new ideas or approaches? ◦ Does it offer a unique perspective or advancement over existing research? ◦ Is there a clear articulation of how this project differs from prior work?
Literature Review and Background	4.31	◦ Does the literature review comprehensively cover relevant prior work? ◦ Is the research gap or need for the proposal justified with sufficient evidence? ◦ How well is the background presented to frame the research question?
Clarity and Coherence	4.46	◦ Are the research problem(s), objectives, and approach well-defined and logically presented? ◦ Does the proposal communicate its goals and methodologies clearly? ◦ Are any technical terms or complex ideas explained clearly?
Significance and Impact	4.54	◦ Does the proposal address a problem of considerable importance to the field or society? ◦ Are the potential outcomes impactful on a scientific, societal, or economic level? ◦ Are the anticipated benefits and advancements clearly stated and realistic?
Methodology and Feasibility	3.69	◦ Is the research design sound, with appropriate methodologies? ◦ Are data collection techniques and analyses described in sufficient detail? ◦ Is the research plan realistic and feasible within the stated timeline and budget?
Qualifications and Expertise of the Research Team	3.77	◦ Does the PI and team have relevant qualifications and track records? ◦ Are the team members’ expertise and roles clearly aligned with the project’s needs? ◦ Are there any partnerships that enhance the project’s expertise or resources?
Research Environment and Resources	3.85	◦ Is the research environment conducive to project success (e.g., facilities, institutional support)? ◦ Are necessary resources (equipment, materials, data access) available and adequate? ◦ Does the institution show a commitment to supporting the project?
Interdisciplinary and Collaborative Approach	4.31	◦ Is there meaningful collaboration across disciplines? ◦ Does the project involve partnerships that bring complementary expertise? ◦ Is there evidence of planned collaboration and communication within the team?
Alignment with National or Institutional Priorities	3.62	◦ Is the project aligned with strategic goals of the institution or country (e.g., public health, sustainability)? ◦ Does the project aim to address national or regional issues? ◦ Does the proposal specify ways it contributes to broader strategic goals?
Public Awareness and Knowledge Transfer	3.62	◦ Are there concrete plans to share findings with relevant stakeholders? ◦ Will the results be communicated in ways accessible to both the scientific and general public? ◦ Are knowledge transfer mechanisms, such as training or publications, outlined?
Research Capacity-Building	4.38	◦ Does the proposal involve training or mentorship for junior researchers or staff? ◦ Are there plans to enhance skills or research capacity at the host institution? ◦ Is there a strategy for building research infrastructure or partnerships?
Budget Justification and Cost-Efficiency	4.00	◦ Is the budget reasonable and aligned with project activities? ◦ Are costs justified in detail and aligned with the goals of the proposal? ◦ Does the proposal demonstrate efficient use of resources?
Data Management and Ethical Standards	3.69	◦ Are there adequate data management and sharing plans? ◦ Does the proposal comply with ethical guidelines (e.g., for human/animal subjects)? ◦ Does the project address issues related to diversity and inclusion?
Sustainability and Long-Term Benefits	3.92	◦ Is there potential for the project’s outcomes to have lasting benefits? ◦ Does the proposal specify plans for sustaining outcomes beyond the funding period? ◦ Are economic, environmental, or social sustainability factors considered?
Innovation and Advancement of Knowledge	3.77	◦ Does the project propose methods or insights that advance the current state of knowledge? ◦ Is there potential for the project to lead to new research opportunities or applications? ◦ Are the proposed contributions unique and impactful?
Outcome Maximisation	3.85	◦ Is there a clear plan to maximize the impact of research outcomes? ◦ Does the project outline stakeholder engagement and result dissemination plans? ◦ Are pathways to impactful results (e.g., policy change, technology development) identified?
Project Timeline and Management	4.46	◦ Is the project timeline realistic and aligned with the proposed activities? ◦ Are project management roles, responsibilities, and schedules clearly defined? ◦ Are contingency plans in place to address potential obstacles?
Impact and Relevance	4.45	◦ Does the project directly address pressing healthcare challenges? ◦ Is the project aligned with institutional or national priorities, such as improving patient care or supporting Oman Vision? ◦ What is the potential impact on system-level improvements or patient outcomes?
Feasibility and Resource Allocation	3.92	◦ Are sufficient resources (financial, technical, human) identified for successful project execution? ◦ Is the timeline realistic, and are logistical aspects well-considered? ◦ Does the project have a clear structure for managing resources effectively?
Bias Mitigation in Decision-Making	3.38	◦ Does the proposal provide evidence, such as preliminary data or feasibility studies, to support the likelihood of achieving the expected results? ◦ Does the proposal present a justified balance between ambitious, innovative goals and the potential for long-term, sustainable impact, demonstrating that resources invested could lead to significant advancements despite inherent risks? ◦ Are limitations and potential conflicts of interest transparently reported, with a commitment to honest reporting of challenges throughout the project?

PI = principal investigator. *Each criterion was rated for suitability on a 5-point Likert scale from 1 (strongly disagree) to 5 (strongly agree). Criteria with a median rating below 3 were eliminated.

A key theme was scalability and replicability, with one participant noting, “Funding should favour projects that can be scaled up or replicated by other researchers, as these efforts contribute to lasting impact and greater dissemination of knowledge.” Another recurring theme was potential societal and economic benefits and applications, particularly for publicly funded projects. One respondent explained, “Projects that demonstrate a potential to benefit society or the economy may be prioritised, especially if aligned with public welfare goals.”

Other highlighted themes included collaborative funding support, with some respondents highlighting the role of international partnerships in augmenting research funding. One participant noted, “If the study will be conducted in Oman and internationally, sometimes the participating international university also funds part of the budget.” Several experts suggested incorporating criteria to assess the transferability of research findings. As one participant remarked, “It may be beneficial to consider adding criteria that assess the transferability of research findings into tangible products, programmes, or initiatives.” Stakeholder and end-user involvement was also highlighted, with one respondent stating, “Including end-users, beneficiaries, or key stakeholders in the planning or design phases enhances the likelihood of practical applications and strengthens societal impact.”

Other suggestions included ensuring context and sociopolitical sensitivity, considering commercialisation potential, and evaluating research maturity for clinical trials. As one respondent pointed out, “The maturity of the research and if it will lead to clinical trials is a key factor in determining its impact on healthcare interventions”.

Qualitative interviews with the three strategic-level decision-makers reinforced the importance of social return on investment (SROI) to evaluate communal benefits, such as improved public health, patient outcomes, and healthcare efficiency, despite challenges in quantification and delayed impact realisation. Innovation was identified as a catalyst for economic growth, with various suggestions proposed, including fostering transdisciplinary collaboration, rewarding diverse teams, and promoting early-career researchers to drive creativity. Experts also stressed the need for collaborative funding through partnerships with external stakeholders, including those in the private sector, to enhance research applicability and ensure a balance between high-risk, high-reward projects and conventional studies.

Sustainability and scalability were also deemed essential, prioritising research with enduring and adaptable benefits across diverse settings. Additionally, experts advocated balancing research and development (R&D)-intensive projects with process improvement initiatives to enhance patient care. Finally, aligning research with local healthcare priorities while maintaining global relevance was viewed as critical for fostering meaningful innovation.

### Phase II: Initial validation

Nine out of 15 experts participated in round 2 of the panel. The evaluation criteria for research proposals were finalised after incorporating expert ratings, averaging scores, and integrating feedback to merge and clarify overlapping categories (Table 3). This process resulted in a final list of 10 criteria.

**Table 3:** Key factors identified by experts when prioritising funding for research projects in Round 2 (n = 9).

Factor	Average Likert score*	Considerations
Scientific Merit and Research Ethics	4.78	◦ Does the literature review cover key prior work and justify the research gap with clear evidence? ◦ Are the research problem(s), objectives, and methods clearly presented? ◦ Is the research design appropriate and well-aligned with objectives? ◦ Are data collection and analysis methods adequately described? ◦ Are ethical concerns addressed, with a plan for protecting participants?
Novelty and Originality	4.78	◦ Is the proposal introducing genuinely new ideas or approaches? ◦ Are the proposed contributions unique and likely to fill a critical knowledge gap?
Significance and Alignment with Institutional, National, and International Goals	4.56	◦ Does the project align with institutional, national, or international goals (e.g., public health, sustainability) and target relevant regional or national issues? ◦ Is it clear how the project contributes to broader strategic objectives and can add to the knowledge in the field?
Innovation and Advancement of Knowledge	4.40	◦ Does the project introduce novel methods or insights that enhance existing knowledge? ◦ Could the project generate new research directions or applications in the field? ◦ Are the proposed contributions distinctively innovative and likely to have a meaningful impact? **◦** Does the project utilize or develop advanced techniques that set it apart from current work? ◦ Does the project foster cross-disciplinary innovation or integrate cutting-edge techniques?
Team Qualifications, Expertise, and Multidisciplinary Approach	4.3	◦ Do the PI and team members have the relevant credentials and proven expertise? ◦ Are team roles clearly defined to ensure each member’s skills align with project needs? ◦ Do partnerships bring additional specialized expertise or resources to the project? ◦ Is the team well-rounded with a mix of disciplines that add value to the project’s objectives?
Interdisciplinary and Collaborative Approach	3.67	◦ Does the project actively integrate expertise from multiple disciplines and sectors (e.g., academia, industry, healthcare, government) to enrich its approach? ◦ Are partnerships established with external organizations that bring complementary skills, resources, and perspectives essential to the project’s success? ◦ Is there a structured plan for regular collaboration, including scheduled meetings, shared objectives, and communication channels to coordinate efforts across disciplines?
Feasibility	4.56	◦ Are all essential resources (equipment, materials, data) accessible and sufficient for project completion? ◦ Does the institution demonstrate strong support for the project, ensuring adequate resources and facilities? ◦ Is the timeline realistic, with detailed planning for resource allocation and activity sequencing? ◦ Are project roles, responsibilities, and contingency plans established to handle potential challenges?
Budget Justification and Cost-Efficiency	4.56	◦ Is the budget proportional to the project’s scope, with a clear rationale for each expense? ◦ Are costs mapped directly to project goals to demonstrate efficient use of funds? ◦ Does the proposal outline strategies for minimizing unnecessary expenses?
Impact	4.67	◦ Does the project have a clear strategy for achieving a significant social return on investment, such as improved public health, economic benefits, or societal well-being? ◦ Are there pathways identified for commercialization, such as developing a new product, service, or technology that could generate revenue or market interest? ◦ Is there potential for the research outcomes to inform policy, industry standards, or guidelines that would create broader societal benefits? ◦ Does the project contribute to capacity-building, such as enhancing research infrastructure or providing mentorship and skill development within the institution? ◦ Are there measurable indicators of impact that align with the strategic priorities of the institution or the broader community?
Public Awareness and Knowledge Transfer	3.56	◦ Are there concrete plans for sharing findings with relevant stakeholders? ◦ Will project results be communicated in formats accessible to both scientific and general audiences? ◦ Are mechanisms for knowledge transfer, like publications, workshops, or training, explicitly outlined? ◦ Does the project include a strategy for engaging the public or key stakeholders in meaningful ways?
Partnerships in Funding	3.40	◦ Has the proposal secured financial support from private sector partners, such as pharmaceutical companies, foundations, or other external organizations? ◦ Are there formal agreements outlining shared risk and accountability, especially valuable for innovative or high-risk projects? ◦ Do partner contributions, whether financial or technical, directly support the project’s objectives and enhance feasibility? ◦ Are the goals of the project aligned with the strategic interests of funding partners, ensuring mutual benefit? ◦ Does the proposal outline a framework for collaboration that includes resource-sharing, risk mitigation, and a commitment to long-term impact?
Sustainability, Scalability, and Replicability	3.89	◦ Are there clear plans for sustaining the project’s outcomes after funding ends? ◦ Does the project consider environmental, social, or economic factors for long-term impact? ◦ Is the approach designed to be adaptable or replicable in different contexts? ◦ Are there defined strategies to maximize the reach and continued relevance of the outcomes?

PI = principal investigator. *Each criterion was rated for suitability on a 5-point Likert scale from 1 (strongly disagree) to 5 (strongly agree). Criteria with a median rating below 3 were eliminated.

The most highly prioritised criteria were *Ethical Standards* and *Scientific Merit* (combined average score: 4.78), highlighting the importance of methodological rigour, ethical integrity, and risk-benefit analyses, and *Novelty and Innovation* (4.78), stressing originality, filling critical knowledge gaps, and scalability. *Significance and Strategic Alignment* (4.56) and *Feasibility and Risk Mitigation* (4.56) were also highly prioritised, ensuring projects are both impactful and realistically executable.

*Impact and Knowledge Transfer* (4.67) focused on the dissemination and the societal and practical benefits of research findings, while *Budget and Cost-Efficiency* (4.56) underscored the need for financial justification, prudent spending, and appropriate resource allocation. *Team Expertise and Collaboration* (4.30) assessed the research team’s capability to execute the project successfully across relevant disciplines. *Sustainability* (3.89) and *Partnerships in Funding* (3.40) addressed long-term viability and external resource contributions.

### Phase III: Weighting and validation

The AHP results prioritized 10 evaluation criteria based on ratings from seven experts. The highest-weighted criterion was *Scientific Merit* (20.16%), indicating it was considered the most critical factor in assessing proposals. This was followed closely by *Ethical Standards* (19.21%) and *Novelty and Innovation* (14.34%), underscoring the importance of methodological soundness and forward-thinking approaches. *Significance and Alignment* (10.10%) and *Team Expertise and Collaboration* (8.39%) also received notable weights, suggesting their perceived influence in the overall impact of the proposal. Other criteria such as *Feasibility and Risk Mitigation* (7.27%), *Impact and Knowledge Transfer* (7.00%), and *Budget and Cost Efficiency* (6.24%) were moderately prioritized. *Sustainability* (4.14%) and *Partnerships in Funding* (3.17%) received the lowest weights, suggesting they were considered less critical in this context.

**Fig 2:**
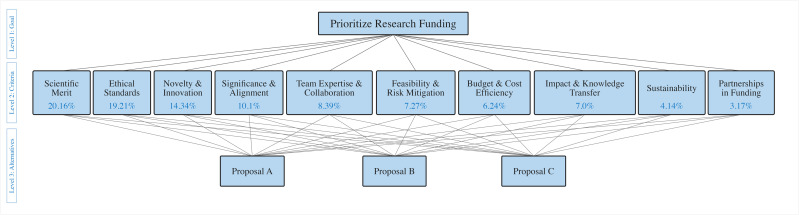
Analytic hierarchy structure for prioritizing research funding.

Each expert provided pairwise comparisons, which were synthesised to generate individual weights and a group consensus score of 0.84586, indicating strong agreement among raters (Table 4). The hierarchical structure of the AHP model effectively captured the relative importance of each criterion in the decision-making framework. This hierarchy not only organises the decision criteria logically but also supports transparent, quantifiable comparisons, reinforcing the credibility of the final prioritisation (Fig 2).

**Table 4:** Key criteria for research funding prioritised by experts using the analytic hierarchy process (n = 7).

	Weighting of each criterion										Consistency ratio
	Scientific Merit	Ethical Standards	Novelty and Innovation	Significance and Alignment	Team Expertise and Collaboration	Feasibility and Risk Mitigation	Budget and Cost Efficiency	Impact and Knowledge Transfer	Sustainability	Partnerships in Funding	
Rater 1	0.116032	0.084609	0.104792	0.115698	0.125357	0.10402	0.128707	0.091525	0.075174	0.054085	0.084923
Rater 2	0.148261	0.21064	0.08612	0.075656	0.087084	0.109287	0.078579	0.0819	0.069398	0.053077	0.152065
Rater 3	0.215673	0.145406	0.117658	0.10338	0.086171	0.079605	0.113504	0.046873	0.060673	0.031056	0.15668
Rater 4	0.348856	0.224073	0.150546	0.093496	0.068888	0.044401	0.028734	0.020131	0.012834	0.008041	0.433839
Rater 5	0.156933	0.224126	0.155613	0.099246	0.103265	0.095298	0.036691	0.064357	0.040057	0.024414	0.242177
Rater 6	0.257305	0.19567	0.148232	0.135818	0.050232	0.043279	0.03377	0.087661	0.021816	0.026217	0.093748
Rater 7	0.179298	0.23591	0.213869	0.064524	0.046452	0.044581	0.042884	0.099335	0.038575	0.034571	0.090522
Group*	0.201562 (20.16%)	0.192083 (19.21%)	0.143399 (14.34%)	0.10101 (10.10%)	0.083879 (8.39%)	0.072654 (7.27%)	0.062351 (6.24%)	0.069991 (7.00%)	0.041416 (4.14%)	0.031655 (3.17%)	0.077499
Consensus score	0.84586										

*The final row shows group averages for each criterion with corresponding percentages.

### Sensitivity analysis: Innovation-focused weighting scenario

This scenario simulated a funding context that places greater emphasis on innovative research while preserving the overall decision structure. The adjusted criterion weights under this scenario are presented in Table 5.

**Table 5:** Adjusted criteria weights under an innovation-focused sensitivity scenario.

Criterion	Original weight (%)	Adjusted weight (%)
Ethical Standards	21.7	20.8
Scientific Merit	19.1	18.3
Novelty & Innovation	16.7	20.0
Significance & Alignment	10.0	9.6
Impact & Knowledge Transfer	8.8	8.4
Feasibility & Risk Mitigation	6.6	6.3
Team Expertise & Collaboration	6.5	6.2
Budget & Cost-Efficiency	4.0	3.8
Sustainability	3.6	3.4
Partnerships in Funding	2.9	2.8
**Total**	**100.0**	**100.0**

In this scenario, the weight assigned to Novelty and Innovation was increased from 16.7% to 20.0%, with proportional reductions applied to the remaining criteria to maintain a total weight of 100%. Comparison of the adjusted and original rankings showed no rank reversal among the top criteria. Ethical Standards and Scientific Merit remained the highest-weighted domains, and the overall hierarchical ordering was preserved.

The limited magnitude of change and absence of structural rank shifts indicate that the prioritization framework is robust to plausible strategic reweighting. This suggests that the model can accommodate shifts in funding emphasis without destabilizing its core decision logic.

## Discussion

This study developed a scoring model to enhance the peer-review process for research funding allocation in healthcare institutions. By establishing 10 key criteria, the model supports transparent, consistent, and impact-driven decision-making, ensuring that resources are directed towards proposals that are not only innovative and scientifically robust but also ethically sound and strategically relevant. Given the challenges of limited funding, diverse evaluation standards, and the need to balance innovation with feasibility, this model integrates expert insights to address these complexities and improve research funding practices.

The identified criteria, which span societal impact, innovation, sustainability, and collaborative funding, reflect a broader perspective on research impact, extending beyond conventional academic metrics. This aligns with growing calls for comprehensive, outcome-focused evaluation frameworks for research that prioritises both local healthcare needs and contributions to global knowledge. The following discussion explores how the model’s key components align with contemporary research funding practices, offer a balanced approach to funding high-impact projects, and provide a foundation for fostering a sustainable, innovation-driven healthcare research culture.

### Refining the peer-review process

Our findings align with existing literature on the limitations and potential refinements of peer-review systems in research funding. Guthrie *et al*. highlight the inherent uncertainties and biases of traditional peer-review processes, including cognitive biases, cronyism, and the challenge of predicting research impact based on bibliometric indicators [19]. These insights reinforce the need for alternative approaches, such as confidence ratings and portfolio-based assessments, to refine peer-review practices and enhance fairness and effectiveness. The researchers advocate for leveraging technological advancements and exploring alternative methodologies like lotteries to complement traditional peer-review processes [19]. Our proposed scoring model aligns with this direction, balancing subjectivity with transparency to refine decision-making.

Predefined selection criteria have been widely recognised for their role in promoting transparency and prioritising impactful research. Tuffaha *et al*. emphasise the importance of criteria such as societal and economic benefits, stakeholder engagement, and alignment with institutional priorities [20]. These elements ensure that funding decisions address pressing real-world healthcare challenges while avoiding redundancy. However, other concepts like cost-effectiveness and systematic reviews are often underutilised in funding decisions, suggesting areas for improvement that could further strengthen the comprehensiveness of our model.

### Evaluating broader impacts and social return on investment

Incorporating SROI as a key evaluation criterion ensures that funding decisions account for long-term societal benefits beyond traditional academic outputs. While publication counts and citation metrics remain dominant, SROI provides a more holistic perspective by considering broader advantages, such as improvements in public health, patient outcomes, and healthcare system efficiency. However, measuring these benefits remains challenging, mainly due to the difficulty quantifying these social impacts within the confines of traditional research frameworks. Moreover, societal benefits often unfold over extended periods of time, making them difficult to assess at the proposal stage.

Despite these complexities, integrating SROI into funding decisions can help prioritise research with tangible public benefits. This approach aligns with recent calls to expand the criteria for assessing research impact, shifting toward models that evaluate both academic contributions and their real-world applications [21]. By embedding SROI within structured scoring frameworks, research institutions can enhance their ability to allocate funds effectively while maximising societal value.

### Innovation as economic catalyst

The global shift toward a knowledge-based economy underscores the importance of fostering innovation in research funding [22]. Our model prioritises transdisciplinary collaboration, recognising that innovation flourishes in diverse, multidisciplinary teams rather than through the efforts of a single principal investigator. Evaluating proposals based on collective expertise rather than individual credentials encourages participation from early-career researchers and facilitates cross-disciplinary idea exchange, strengthening the research ecosystem.

Studies indicate that deep-level diversity in culturally varied teams enhances creativity and innovation, particularly in interdependent tasks requiring collaborative problem-solving [23]. By valuing team-based approaches, our model promotes inclusivity, nurtures creativity, and ensures that innovative ideas receive the necessary support to progress from conceptualisation to implementation.

### Collaborative funding and risk mitigation

High-impact research often entails financial and operational risks, necessitating collaborative funding strategies to distribute these burdens. Our model incorporates partnerships with external stakeholders, including private-sector entities, to enhance research applicability and financial resilience. Literature on collaborative funding highlights its role in risk mitigation and accelerating the translation of research into practical applications [24].

Engaging industry partners not only expands funding opportunities but also strengthens pathways for commercialisation, fostering a balanced research environment where high-risk, high-reward projects can coexist with more conventional studies. This balanced approach supports a diverse research portfolio, ensuring a sustainable and adaptive innovation ecosystem.

### Sustainability and scalability

Sustainability is a fundamental consideration in healthcare research, where long-term impact is as crucial as immediate outcomes. Our model prioritises projects that demonstrate sustainability, ensuring that funded research not only addresses immediate healthcare and societal needs but also possesses the potential to be expanded effectively across different settings. This aligns with the increasing emphasis in funding literature on sustainable research practices that extend beyond individual projects to influence broader healthcare systems [25].

By supporting proposals with the potential for enduring, scalable benefits, our model maximises SROI and reinforces a resilient research culture that prioritises long-term knowledge advancement. Scalability not only extends the reach and relevance of research outcomes but also enhances healthcare systems’ ability to adapt to emerging challenges.

### Balancing research and development with process improvement

While technological advancements remain a core focus of many funding models, our approach acknowledges the equal importance of research aimed at improving healthcare processes. Process-oriented research has been shown to enhance patient care and system efficiency, contributing significantly to healthcare outcomes without necessarily resulting in new technologies [26]. By accommodating both R&D-intensive projects and process-driven improvements, our model ensures a well-rounded funding strategy that strengthens the healthcare sector’s resilience and adaptability.

### Context sensitivity and global relevance

Ensuring that funded research aligns with local healthcare priorities while maintaining global relevance enhances both the applicability and impact of research outcomes. Context sensitivity has been identified as a key factor in increasing the likelihood of research generating direct benefits [27]. Our model encourages projects that address pressing national healthcare challenges while contributing to the global knowledge base, fostering innovation that serves both Oman and the international community.

By integrating structured evaluation criteria, this study contributes to ongoing efforts to refine research funding mechanisms. Implementing such models enhances funding equity, promotes impactful research, and ensures that resources are allocated towards projects with the greatest potential for societal and scientific benefit.

### Plans for model implementation

A phased implementation approach will be adopted to ensure the effective integration of the proposed scoring model. An initial pilot testing phase will be conducted at the Royal Hospital to compare the model’s effectiveness against traditional peer-review methods, followed by structured reviewer training to standardise evaluation practices. The model will then be embedded into the hospital’s funding framework through an online platform facilitating systematic scoring and proposal evaluation.

To promote broader adoption, national and international cross-institutional collaborations will be encouraged, aligning the model with best practices in research funding. Additionally, AI-artificial intelligence (AI)-driven decision-support tools will be integrated to optimise the process, enabling automated initial screenings, predictive impact assessments based on historical data, and bias detection in peer reviews. Natural language processing algorithms will further enhance objectivity, reducing reviewer fatigue while improving transparency.

A systematic review and update process will be established to ensure the model’s long-term relevance and effectiveness. Annual stakeholder evaluations, data-driven assessments of funded project outcomes, and adaptive learning mechanisms using AI analytics will refine selection criteria based on emerging research trends. Benchmarking against international funding frameworks will facilitate continuous improvement and alignment with best practices. Expanding the model’s application beyond the Royal Hospital and leveraging digital tools will be key priorities in enhancing research funding fairness, efficiency, and impact. By adopting these measures, healthcare institutions can maximise research investments, fostering meaningful medical innovation and advancing patient care.

This study has several limitations. First, although expert input was obtained from diverse professional backgrounds, the weighting process was conducted within a single institutional context, which may limit the generalisability of the findings to other funding environments. Second, while structured methods such as the Delphi technique and the Analytic Hierarchy Process were used to enhance transparency and consistency, the prioritisation framework remains dependent on expert judgment. Third, the sensitivity analysis focused on assessing rank stability under alternative weighting scenarios rather than full numerical recomputation of AHP matrices. Finally, the study did not evaluate the long-term impact of implementing the proposed tool in real-world funding decisions, and future research should assess its performance through longitudinal application.

## Conclusion

This study developed a structured and transparent scoring model to improve the peer-review process for research funding allocation in healthcare institutions. The final framework comprised ten weighted criteria, with ethical standards (21.7%), scientific merit (19.1%), and novelty and innovation (16.7%) emerging as the most influential. These findings demonstrate the utility of a structured, transparent approach to improving consistency and accountability in research funding decisions.

By incorporating expert insights, stakeholder feedback, and a systematic weighting approach using AHP, the model addresses key challenges such as subjectivity, inconsistency, and bias in funding decisions. The findings underscore the importance of ethical standards, scientific merit, and innovation in prioritising research proposals, ensuring that resources are directed towards high-impact projects with meaningful contributions to scientific advancement and healthcare improvement. Implementing this model has the potential to enhance the fairness and efficiency of research funding processes, fostering a culture of evidence-based decision-making in healthcare research funding allocation.

## Supporting information

S1 Appendix Appendix 1: Final list of criteria determined for a peer-review decision support tool for research funding. (DOCX)

## Abbreviations

AHP: Analytic Hierarchy Process
AI: Artificial Intelligence
PI: Principal Investigator
R&D: Research and Development
SROI: Social Return on Investment
TRC: The Research Council (Oman)
SQU: Sultan Qaboos University
NIH: National Institutes of Health
RIMA: Southern African Research and Innovation Management Association

## References

[1] Williams K, Grant J. A comparative review of how the policy and procedures to assess research impact evolved in Australia and the UK. Res Eval. 2018;27:93–105. doi: 10.1093/reseval/rvx042

[2] Kok MO, Schuit AJ. Contribution mapping: a method for mapping the contribution of research to enhance its impact. Health Res Policy Syst. 2012;10:21. doi: 10.1186/1478-4505-10-21 22748169 PMC3464695

[3] Resnik DB. Practical problems related to health research funding decisions. Am J Bioeth. 2018;18(11):21–2. doi: 10.1080/15265161.2018.1523494 30976205 PMC6452879

[4] Association of Medical Research Charities. Reviewing peer review. [cited 2025 Mar 2]. Available from: https://www.amrc.org.uk/pages/faqs/category/reviewing-peer-review

[5] Shepherd J, Frampton GK, Pickett K, Wyatt JC. Peer review of health research funding proposals: a systematic map and systematic review of innovations for effectiveness and efficiency. PLoS One. 2018;13(5):e0196914. doi: 10.1371/journal.pone.0196914 29750807 PMC5947897

[6] Gallo SA, Carpenter AS, Glisson SR. Teleconference versus face-to-face scientific peer review of grant application: effects on review outcomes. PLoS One. 2013;8(8):e71693. doi: 10.1371/journal.pone.0071693 23951223 PMC3740535

[7] van Bekkum JE, Hilton S. UK research funding bodies’ views towards public participation in health-related research decisions: an exploratory study. BMC Health Serv Res. 2014;14:318. doi: 10.1186/1472-6963-14-318 25056498 PMC4115156

[8] Barnett AG, Herbert DL, Campbell M, Daly N, Roberts JA, Mudge A, et al. Streamlined research funding using short proposals and accelerated peer review: an observational study. BMC Health Serv Res. 2015;15:55. doi: 10.1186/s12913-015-0721-7 25888975 PMC4324047

[9] Abdoul H, Perrey C, Amiel P, Tubach F, Gottot S, Durand-Zaleski I, et al. Peer review of grant applications: criteria used and qualitative study of reviewer practices. PLoS One. 2012;7(9):e46054. doi: 10.1371/journal.pone.0046054 23029386 PMC3460995

[10] Street J, Baum F, Anderson IPS. Is peer review useful in assessing research proposals in Indigenous health? A case study. Health Res Policy Syst. 2009;7:2. doi: 10.1186/1478-4505-7-2 19216770 PMC2654449

[11] Goldstein A, Kearney M. Uncertainty and individual discretion in allocating research funds. Working paper 32033. Cambridge (MA): National Bureau of Economic Research; 2024.

[12] Gallo SA, Sullivan JH, Glisson SR. The influence of peer reviewer expertise on the evaluation of research funding applications. PLoS One. 2016;11(10):e0165147. doi: 10.1371/journal.pone.0165147 27768760 PMC5074495

[13] Fang FC, Bowen A, Casadevall A. NIH peer review percentile scores are poorly predictive of grant productivity. Elife. 2016;5:e13323. doi: 10.7554/eLife.13323 26880623 PMC4769156

[14] Mayo NE, Brophy J, Goldberg MS, Klein MB, Miller S, Platt RW, et al. Peering at peer review revealed high degree of chance associated with funding of grant applications. J Clin Epidemiol. 2006;59(8):842–8. doi: 10.1016/j.jclinepi.2005.12.007 16828678

[15] SciVal. Institution overview: Royal Hospital Oman. Elsevier. [cited 2025 Mar 2]. Available from: https://www.scival.com/overview/summary?uri=Institution/717229

[16] Saaty RM. The analytic hierarchy process—what it is and how it is used. Math Model. 1987;9:161–76. doi: 10.1016/0270-0255(87)90473-8

[17] Whitaker R. Validation examples of the analytic hierarchy process and analytic network process. Math Comput Model. 2007;46:840–59. doi: 10.1016/j.mcm.2007.03.018

[18] Meixner O. Fuzzy AHP group decision analysis and its application for the evaluation of energy sources. Cent Eur J Oper Res. 2009;17:347–69. doi: 10.13033/isahp.y2009.051

[19] Guthrie S, Ghiga I, Wooding S. What do we know about grant peer review in the health sciences? F1000Res. 2017;6:1335. doi: 10.12688/f1000research.11917.2 29707193 PMC5883382

[20] Tuffaha HW, El Saifi N, Chambers SK, Scuffham PA. Directing research funds to the right research projects: a review of criteria used by research organisations in Australia in prioritising health research projects for funding. BMJ Open. 2018;8(12):e026207. doi: 10.1136/bmjopen-2018-026207 30580278 PMC6318516

[21] Ashton K, Cotter-Roberts A, Dyakova M, Green L. Integration of social return on investment with health impact assessment and evaluation frameworks. Eur J Public Health. 2023;33(Supplement_2):ckad160.1285. doi: 10.1093/eurpub/ckad160.1285

[22] Yu KE. Global trends and tendencies of becoming knowledge economy in the world economy. Sci Notes V I Vernadsky Crimean Fed Univ Econ Manag. 2020;6(72)(2):59–74. doi: 10.37279/2413-1644-2020-6-2-59-74

[23] Wang J, Cheng GHL, Chen T, Leung K. Team creativity/innovation in culturally diverse teams: a meta-analysis. J Organ Behav. 2019;40:693–708. doi: 10.1002/JOB.2362

[24] Vasan K, West JD. The hidden influence of communities in collaborative funding of clinical science. R Soc Open Sci. 2021;8(8):210072. doi: 10.1098/rsos.210072 34457332 PMC8385381

[25] Scheirer MA. Linking sustainability research to intervention types. Am J Public Health. 2013;103(4):e73-80. doi: 10.2105/AJPH.2012.300976 23409904 PMC3673273

[26] Kohlbacher M. The effects of process orientation: a literature review. Bus Process Manag J. 2010;16(1):135–52. doi: 10.1108/14637151011017985

[27] Barbara J, Haley N, McMahon H, Turnbull T. Building inherently impactful research programs: the role of organizational context. Policy Des Pract. 2021;4(3):357–71. doi: 10.1080/25741292.2021.1946246

