# Supplementary material for: A peer-review decision support tool for research funding decision-making using the analytic hierarchy process
Source: PLoS One. 2026 Aug 12;21(8):e0350938. doi: 10.1371/journal.pone.0350938 (PMC13465840; doi:10.1371/journal.pone.0350938)
Supplement: Supplementary file 1 [file pone.0350938.s001.docx]

**Appendix 1:** Final list of criteria determined for a peer-review decision support tool for research funding

| **Criteria** | **Definition** | **Guiding Questions** |
| --- | --- | --- |
| **1. Scientific Merit** | The proposal is methodologically sound, with strong rigor of the research design, including its foundation in existing literature and alignment between objectives and methods. | - Literature Review:   - Does the review comprehensively cover prior work and justify the research gap with clear evidence? - Research Problem, Objectives, and Approach:   - Are the problem, objectives, and methods well-defined and logically presented? - Research Design and Methodology:   - Is the design sound and aligned with the objectives?   - Are data collection and analysis methods clearly described?   - Does the project have defined interim milestones and a mechanism for monitoring progress? |
| **2. Ethical Standards** | Research ethical issues are well-addressed, with a clear risk-benefit assessment. | - Are ethical concerns adequately addressed? - Is there a robust plan to protect participants? - Does the proposal include a risk-benefit analysis to ensure the benefits outweigh potential risks? |
| **3.** **Novelty and Innovation** | The project introduces original ideas, methods, or applications and has the potential for transformative research. | - Does the project introduce genuinely new ideas or methods? - Are contributions unique and likely to fill a critical knowledge gap? - Could the project foster new research directions, cross-disciplinary approaches, or cutting-edge techniques? - Is the project scalable or adaptable to different populations or contexts? |
| **4. Significance and Strategic Alignment** | The research aligns with institutional or national priorities and addresses important societal needs. | - Institutional and National Goals:   - Does the project align with strategic priorities like public health or sustainability?   - Does it address relevant social and institutional issues? - Broader Impact:   - Does it contribute to global research priorities, such as the Sustainable Development Goals (SDGs)?   - Does it actively engage stakeholders (e.g., patients, policymakers, communities) in the design or execution phase? |
| **5. Team Expertise and Collaboration** | The research team is qualified, and collaborations enhance the project’s quality and scope. | - Team Composition:   - Do team members have relevant qualifications and expertise?   - Are roles clearly defined? - Partnerships and Multidisciplinary Integration:   - Are partnerships with external organizations established?   - Does the collaboration enhance the project with diverse expertise? - Coordination:   - Is there a structured plan for regular communication and shared objectives? |
| **6. Feasibility and Risk Mitigation** | The project is realistic and executable, given the available resources and institutional support, and includes clear plans to manage potential risks. | - Are necessary resources accessible, and does the institution provide adequate support? - Is the timeline realistic, with contingency plans to address potential challenges? - Is the risk mitigation plan clear and well-defined? |
| **7. Budget and Cost Efficiency** | The budget is justified, cost-effective, and aligned with project goals. | - Is the budget justified and proportional to the scope? - Are expenses clearly linked to project goals? - Is the budget required for tasks beyond data collection and research assistance? - Does the proposal minimize unnecessary costs and include external financial support where applicable? |
| **8. Impact and Knowledge Transfer** | The research has clear potential for societal, economic, or policy impact and includes strong plans for sharing results. | - Social and Economic Impact:   - Does the project have measurable societal benefits or commercialization potential? - Capacity-Building and Education:   - Does it enhance institutional research capacity and mentorship?   - Are there plans for training and workshops during the project lifecycle? - Dissemination:   - Are there clear plans for sharing findings with stakeholders through publications, workshops, or accessible formats? - Policy and Standards:   - Can the research outcomes inform policies, industry standards, or guidelines? |
| **9. Sustainability** | Outcomes are likely to continue beyond the project, with long-term social, economic, or environmental benefits. | - Are there clear plans for sustaining outcomes beyond the funding period? - Does the project consider environmental, social, and economic factors for long-term impact? - Is the approach designed to be replicable and adaptable in different contexts? |
| **10. Partnerships in Funding** | The project includes complementary funding or support that enhances feasibility and reduces financial risk. | - Does the research team have additional funding sources (national or international) to complement the requested budget? - Could these funding partnerships lower the financial risk, especially for innovative ideas with a higher risk of failure compared to traditional research? - Do partner contributions (financial or technical) directly support the project’s objectives and enhance feasibility? |
